# Cyclic nucleotide signaling in sensory neuron hyperexcitability and chronic pain after nerve injury

**DOI:** 10.1016/j.ynpai.2019.100028

**Published:** 2019-03-08

**Authors:** Ze-Hua Li, Dong Cui, Cheng-Jie Qiu, Xue-Jun Song

**Affiliations:** a Department of Biology, SUSTech Center for Pain Medicine, and Medical School, Southern University of Science and Technology, Shenzhen, Guangdong 518055, China; b Department of Anesthesiology and Key Laboratory of Carcinogenesis and Translational Research (Ministry of Education of China), Peking University School of Oncology, Beijing Cancer Hospital & Institute, Beijing 100142, China

## Abstract

The cyclic nucleotide signaling, including cAMP-PKA and cGMP-PKG pathways, has been well known to play critical roles in regulating cellular growth, metabolism and many other intracellular processes. In recent years, more and more studies have uncovered the roles of cAMP and cGMP in the nervous system. The cAMP and cGMP signaling mediates chronic pain induced by different forms of injury and stress. Here we summarize the roles of cAMP-PKA and cGMP-PKG signaling pathways in the pathogenesis of chronic pain after nerve injury. In addition, acute dissociation and chronic compression of the dorsal root ganglion (DRG) neurons, respectively, leads to neural hyperexcitability possibly through PAR2 activation-dependent activation of cAMP-PKA pathway. Clinically, radiotherapy can effectively alleviate bone cancer pain at least partly through inhibiting the cancer cell-induced activation of cAMP-PKA pathway. Roles of cyclic nucleotide signaling in neuropathic and inflammatory pain are also seen in many other animal models and are involved in many pro-nociceptive mechanisms including the activation of hyperpolarization-activated cyclic nucleotide (HCN)-modulated ion channels and the exchange proteins directly activated by cAMP (EPAC). Further understanding the roles of cAMP and cGMP signaling in the pathogenesis of chronic pain is theoretically significant and clinically valuable for treatment of chronic pain.

## Highlights

• Activation of cAMP-PKA and cGMP-PKG pathways contributes to injury-induced sensory neuron hyperexcitability.
• Activation of cAMP and cGMP contributes to the development of bone cancer pain.
• PAR2 activation mediates injury-induced cAMP-dependent sensory neuron hyperexcitability.

## 1 Overview of cAMP-PKA and cGMP-PKG pathways

Cyclic adenosine monophosphate (cAMP)-protein kinase A (PKA) pathway is initiated by the binding of an extracellular ligand to G protein coupled receptor (GPCR). GPCR consists of two functional structures: extracellular pocket for ligand recognition and intracellular cleft for interaction with membrane-bound heterotrimeric G proteins. Heterotrimeric G protein is a complex made up of three subunits, alpha (α), beta (β) and gamma (γ). Once the GPCR is activated, the α subunit of stimulatory G protein (G_s_) dissociates from the βγ complex and promotes the activity of adjacent adenylate cyclase (AC), which then catalyzes the conversion from ATP to cAMP and ultimately increases cAMP concentration in the cytosol. Cytoplasmic cAMP serves as a second messenger which activates its sensors including the widely known protein kinase PKA, as well as the exchange proteins directly activated by cAMP (EPAC), the cyclic nucleotide regulated ion channels and the Popeye domain containing (POPDC) proteins (Zufall et al., 1997, Kawasaki et al., 1998, de Rooij et al., 1998, Krahling et al., 2013, Schindler and Brand, 2016). The classic target of cAMP, PKA further catalyzes phosphorylation of other proteins and causes a series of downstream changes (Nelson and Cox, 2008). PKA, the core enzyme in this pathway, is a holoenzyme complex composed of two regulatory subunits (PKA-R) and two catalytic subunits (PKA-C). Two types of regulatory subunits have been identified: PKA-RI, mutations of which lead to alternations in inflammation responses and nociceptive pain (Goodwin et al., 1997, Malmberg et al., 1997); PKA-RII, which actively participates in the N-methyl-d-aspartate (NMDA)-dependent synaptic plasticity (Yang et al., 2009, Li et al., 2001, Zhuo et al., 2011). The regulatory and catalytic activity of PKA holoenzyme complex is regulated by scaffolding proteins known as A-kinase anchoring protein (AKAP), which anchor the catalytic subunits to its target molecules or organelles (Alto et al., 2002, Langeberg and Scott, 2005). PKA-RII is able to bind to most of the A-kinase anchoring proteins identified so far preferentially (Rathee et al., 2002).

Activation of the cAMP-PKA pathway is widely reported to have significant effects on many essential cellular and biological processes such as immune function (Serezani et al., 2008), growth (Stork and Schmitt, 2002), differentiation (Yamamizu and Yamashita, 2011), and metabolism (Holz et al., 2008). More and more studies have gradually uncovered the vital functions of cAMP-PKA pathway in the nervous system including synaptic plasticity (Waltereit and Weller, 2003), a prime mechanism underlying chronic pain (Luo et al., 2014). It has been reported that the cAMP-PKA pathway contributes to both early and late phase of initiation of LTP in mossy fibers (Huang et al., 1994). In the hippocampus of transgenic mice that express R (AB), an inhibitory form of the PKA regulatory subunit, the late phase of LTP in CA1 region and related long-term memory can be significantly suppressed compared with naïve animals (Abel et al., 1997). Inhibitors of PKA result in blockade of late component of LTP (L-LTP) while the analogs of cAMP induce potentiation that facilitate L-LTP (Frey et al., 1993). This indicates the crucial role of the cAMP-PKA pathway in the induction and maintenance of synapse plasticity in the nervous system. Activated cAMP-PKA pathway also promotes the synthesis of presynaptic neurotransmitters and vesicular transport by phosphorylating key transcription factors such as cAMP response element-binding protein (CREB) and synaptic vesicle proteins such as snapin (Koppert, 2004, King et al., 2005, Tumati et al., 2011). Studies have demonstrated the involvement of cAMP-PKA pathway in inflammatory pain (Malmberg et al., 1997, Hingtgen et al., 1995, Lewin and Walters, 1999), neuropathic pain (Song et al., 2006, Zheng et al., 2007, Huang et al., 2012) and bone cancer pain (Zhu et al., 2014, Zhu et al., 2016).

The cGMP-PKG pathway is another crucial cyclic nucleotide signaling. The production of cGMP is catalyzed by guanylyl cyclases (GCs), which consist of two types, soluble guanylyl cyclase (sGC) and membrane bound guanylyl cyclase (mGC) and are degraded by cyclic nucleotide phosphodiesterase (PDE). sGC can be an immediate downstream effector of nitric oxide (NO) and is involved in many physiological conditions such as blood pressure regulation, wound healing and memory formation (Montfort et al., 2017). Protein kinase G (PKG) is a serine/threonine kinase activated by cGMP. cGMP also has two other main targets, cyclic-nucleotide gated channel (CNGC) and PDE (Malbon, 2005, Maurice et al., 2014). cGMP-PKG pathway is reported to be important to the guidance and connectivity of sensory axons during development (Schmidt et al., 2002, Song et al., 1998). The cGMP-PKG pathway also regulates synaptic plasticity and two main mechanisms have been hypothesized. First, NMDA receptor can increase the quantity and activity of cGMP through nitric oxide synthase (NOS) pathway and further activate cGMP-dependent protein kinase type II (cGKII), which phosphorylates aminomethylphosphonic acid (AMPA) receptor subunit GluA1 and leads to increased expression of AMPA receptor on extrasynaptic membrane sites (Serulle et al., 2007). Consistently, the cGKII-knockout mice exhibited weaker abilities in spatial learning compared to controls (Wincott et al., 2013). Secondly, cGMP may contribute to the retrograde modulation by responding to NO release from postsynaptic myocytes. NO may further activate cGMP in presynaptic terminals and thus suppress its spontaneous and evoked synaptic currents in developing neuromuscular synapses (Wang et al., 1995). Earlier reports have also shown that cGMP enhances neurotransmitter release and activates CNGC to enable Ca^2+^ to enter (Savchenko et al., 1997). These activations ultimately enhance the Ca^2+^-triggered release of neurotransmitters in hippocampal neurons (Arancio et al., 1995) and in a Ca^2+^-independent manner in motoneurons and ciliary ganglion neurons of Drosophila larval (Wildemann and Bicker, 1999, Yawo, 1999). Studies have also demonstrated the roles of cGMP signaling in the pathogenesis of neuropathic pain (Song et al., 2006, Huang et al., 2012).

Previous studies have also highlighted the cross-talk between cAMP-PKA and cGMP-PKG pathway, especially in the context of cerebral vasodilation (Pelligrino and Wang, 1998). Accumulated evidence has indicated that cAMP-PKA and cGMP-PKG pathway can work in concert to enhance LTP, implicating improvement of long-term memory and development of pain (Matsumoto et al., 2006, Bollen et al., 2014).

## 2 Activation of cAMP-PKA and cGMP-PKG pathway contributes to sensory neuron hyperexcitability and behavioral hyperalgesia in rats with DRG compression

Injury of DRG sensory neurons and associated inflammation are common causes of neuropathic pain (Devor, 1999, Zimmermann, 2001; Abdulla and Smith, 2001, Bennett and Xie, 1988, Hu and Xing, 1998, Ma and LaMotte, 2005, Song et al., 1999, Song et al., 2003a, Song et al., 2003b, Stebbing et al., 1999, Wall and Gutnick, 1974, Zhang et al., 1999). The electrophysiological alterations (Rizzo et al., 1995, Waxman, 1999, Everill and Kocsis, 1999, Yao et al., 2003, Abdulla and Smith, 2001, Baccei and Kocsis, 2000) and possible transcriptional changes (Costigan et al., 2002, Valder et al., 2003, Xiao et al., 2002) within DRG neurons may underlie neural hyperexcitability induced by different forms of stress. Nevertheless, the intracellular mechanisms underlying these alterations still remain elusive. Considerable evidence has indicated that cAMP-PKA pathway can be activated by prostaglandin E2 (PGE2) in isolated neurons and plays key roles in the alternations of electrophysiological properties of DRG neurons that ultimately result in the increased neuronal excitability (Hingtgen et al., 1995, Fowler et al., 1985, England et al., 1996, Gold et al., 1996, Ingram and Williams, 1996, Caterina et al., 1997, Lopshire and Nicol, 1998, Evans et al., 1999). In addition to the increase of short-term neuronal excitability, activation of cAMP-PKA pathway after nerve injury can lead to nuclear translocation of PKA-dependent signals, which causes long-lasting alterations in protein synthesis through regulating transcription of specific genes. This process is considered important to synapse plasticity, which is crucial in certain types of learning and memory (Kandel, 2013) and in spinal central sensitization (Malmberg et al., 1997, Ma et al., 2003, Miletic et al., 2002). Besides, it was reported that continuing activation of cAMP-PKA pathway can maintain sensory neuron hyperexcitability and behavioral hyperalgesia (Aley and Levine, 1999, Aley and Levine, 2002, Bolyard et al., 2000, Liao et al., 1999). However, much less attention has been paid to its role in neuropathic pain (Hingtgen et al., 1995, Fowler et al., 1985, England et al., 1996, Gold et al., 1996, Caterina et al., 1997, Lopshire and Nicol, 1998, Evans et al., 1999, Aley and Levine, 1999, Akins and McCleskey, 1993, Cui and Nicol, 1995, Taiwo et al., 1989).

Chronic compression of DRG (CCD treatment) simulates a pathological process in many diseases like lumbar disc protrusion (Song et al., 2006). CCD has been used as an animal model for chronic pain caused by nerve compression. During CCD treatment, lumbar ganglia are compressed by a stainless steel rod inserted into the intervertebral foramen to produce long-lasting chronic pain. CCD rats show significant thermal and mechanical hyperalgesia/allodynia and develop varying degrees of abnormality in gait and posture, indicating spontaneous painful conditions. CCD treatment-induced increased spontaneous activity of DRG neurons with myelinated axons that can be prevented by the treatment of specific inhibitors of PKA (Hu et al., 2001). Multiple pharmacological and electrophysiological studies have identified the involvement of PKA in the regulation of CCD-induced electrophysiological alterations (Hu et al., 2001) and consequent formation of peripheral and central sensitization that leads to thermal hyperalgesia and mechanical allodynia (Liu et al., 2000, Ma and Woolf, 1996, Mannion et al., 1999, Neumann et al., 1996). Further studies have confirmed that DRG compression can activate both cAMP-PKA and cGMP-PKG pathways and induce DRG neuron hyperexcitability. CCD treatment can promote cAMP concentration, as well as mRNA and protein level of PKA-RII and PKA-c in the spinal cord and DRG in rats (Huang et al., 2012). The level of cGMP, type 1 PKG (PKG-I) mRNA and PKG-I protein are also elevated, suggesting the activation of cGMP-PKG signaling after DRG compression (Huang et al., 2012). In terms of electrophysiology, CCD DRG neurons exhibit hyperexcitability and somata depolarization, manifested as spontaneous ectopic discharges, decreased action potential (AP) current threshold and accommodation (also termed spike frequency adaptation) and enhancement of repetitive discharges. More specifically, CCD treatment causes decreased AP voltage thresholds in DRG neurons while increased AP duration in medium- and small-sized ones (Song et al., 2006). Different types of membrane-permeant specific activators or inhibitors of the cAMP-PKA and cGMP-PKG pathways can significantly increase or decrease DRG neurons hyperexcitability after CCD treatment, but had little influence on somata depolarization, which may stem from that CCD treatment has already depolarized resting membrane potential (RMP). Despite the cross-activation between cAMP-PKA and cGMP-PKG pathways in certain tissues (Jiang et al., 1992), inhibitors with high selectivity of either of these pathways could significantly increase AP threshold current and reduce repetitive discharge, suggesting that activation of cAMP-PKA and cGMP-PKG pathways may contribute to the DRG neuron hyperexcitability. CCD DRG neurons become more hyperexcitable and exhibit increased responsiveness to the activators of cAMP-PKA and cGMP-PKG pathways. Consistent with these alternations of the electrophysiological results, inhibitor of either cAMP-PKA or cGMP-PKG pathway applied into the intervertebral foramen greatly suppressed thermal hyperalgesia induced by CCD (Song et al., 2006). This provides key evidence that the cAMP-PKA and cGMP-PKG pathways may contribute to sensory neuron hyperexcitability and the development of hyperalgesia in rats with DRG chronic compression. Inflammation and synthesis of ion channels have been considered to play important roles in DRG neural hyperexcitability and their processes are relatively slow (Waxman, 1999, Ma et al., 2006, Ishikawa et al., 1999, Kim et al., 2002, Waxman et al., 1994, Waxman et al., 2000, Waxman et al., 1999, Zhang et al., 1997). However, it was found that inhibitors of cAMP-PKA or cGMP-PKG pathway administered into the intervertebral foramen could result in rapid attenuation of the hyperexcitability of CCD somata (Song et al., 2006), indicating that DRG neuron hyperexcitability depends, at least partly, on continuing activation of cAMP-PKA pathway.

As for cGMP-PKG pathway, studies have provided evidence for the participation of cGMP and PKG in the induction of hyperalgesia in the spinal cord rather than in DRG (Meller and Gebhart, 1993, Niedbala et al., 1995, Salter et al., 1996, Schmidtko et al., 2003, Tegeder et al., 2004). On the other hand, the activation of cGMP-PKG pathway in DRG neurons has been believed to have depressive (Duarte et al., 1992, Kress et al., 1996, Liu et al., 2004, Sachs et al., 2004) rather than sensitizing effects (Tegeder et al., 2004, Aley et al., 1998, Parada et al., 2005, Vivancos et al., 2003). However, activation of cGMP-PKG pathway in compressed DRG may exert sensitizing effects in the condition of CCD treatment. Neuronal excitability is increased with the activation of cGMP-PKG pathway in compressed DRG that is similar to what is seen in dissociated DRG neurons (Liu and Simon, 2003, Pollock et al., 2003). Several types of hyperalgesia require peripheral NO release to stimulate local cGMP synthesis and mediate certain neuronal processes in DRG (Aley et al., 1998). There is a possibility that CCD treatment may lead to release of cytokines and inflammatory mediators and such signals may activate NO synthesis and enhance cGMP synthesis (Bauer et al., 1995, Hess et al., 1993), which then mediate neuronal hyperexcitability via the NO-cGMP-PKG signaling axis (Ding et al., 2010, Wang et al., 2015). It is believed that NO production is dependent on the Ca^2+^ influx and the subsequent activation of NF-κB. These studies established a model of cGMP signaling in injury-induced DRG neuron hyperexcitability and pain process (Wang et al., 2011).

Despite agonists for cAMP and cGMP pathways showed poor effects on RMP of the CCD DRG neurons, they exert positive effects on the neuronal hyperexcitability. Surprisingly, activation of these pathways in DRG neurons from naive rats led to significant depolarization. Meanwhile, antagonists of these pathways fail to affect the RMP and the excitability of DRG neuron from naive rats (Song et al., 2006). These results suggest that activation of both cAMP and cGMP pathways can depolarize RMP without affecting the excitability of DRG neurons from naïve rats, and neither of the pathways contributes directly to the RMP or excitability in naïve DRG neurons. The little influence of cAMP-PKA pathway on neuronal hyperexcitability in naïve rats seems to be contrary to many of the earlier reports. The most possible explanation is the differences in pretreatments of those neurons. These studies used either somata of dissociated DRG neurons (Rathee et al., 2002, England et al., 1996, Gold et al., 1996, Ingram and Williams, 1996, Caterina et al., 1997, Lopshire and Nicol, 1998, Evans et al., 1999, Akins and McCleskey, 1993, Cui and Nicol, 1995, Aley et al., 1998, Smith et al., 2000, Cardenas et al., 2001) or previously compressed neurons in excised ganglia (Hu et al., 2001), in contrast of the sensory neuron somata that remain within excised but intact ganglia used in the aforementioned investigations. Since the cAMP-PKA and cGMP-PKG pathways have positive effects on hyperexcitability in CCD neurons, the sensitizing effect on sensory neurons of these two pathways requires former injury-related stress, which is produced in these cases by either acute dissociation or chronic compression, the CCD treatment. It is suggested that different forms of stress such as chronic compression and acute dissociation of DRG neurons can increase neuronal electrophysiological responsiveness to the cAMP-PKA and cGMP-PKG pathways, which further mediate the maintenance of sensory neuronal hyperexcitability persistently (Song et al., 2006).

The potential molecular mechanisms underlying this pathological phenomenon are intriguing, which differ a lot from the physiological state. The cAMP-PKA and cGMP-PKG pathways play significant roles in various physiological processes. There has been evidence for the cross-talk between these two signaling pathways. For example, cardiovascular studies found that cAMP-PKA-mediated signaling participated in the response to fight-or-flight in physiological state and continuing activation of this signaling might result in heart failure, while sustained activation of cGMP-mediated signaling has vasodilatory and anti-hypertrophic effects (Weber et al., 2017). In nervous system, the cGMP-PKG pathway tends to excite most of the identified pyramidal tract neurons while the cAMP-PKA pathway always has depressive effects in mammalian motor cortex (Stone and Taylor, 1977). During the development process of nervous system, the cAMP-PKA pathway leads to the initiation of axonal differentiation and stabilization (Shelly et al., 2007) while the cGMP-PKG pathway favors the differentiation of immature neurites into mature dendrites (Shelly et al., 2010). In accordance with the close relationship between cAMP-PKA and cGMP-PKG pathways, there are complicated molecular mechanisms regulating the cross-talk between these two pathways, including activities of PDE, cGMP/cAMP, PKA/PKG as well as compartmentalization of cAMP and cGMP pathways (Pelligrino and Wang, 1998).

cAMP-PKA pathway and cGMP-PKG pathway may produce antagonistic effects under physiological state (McGehee et al., 1992, Dontchev and Letourneau, 2003), and are normally cross-linked to excitability mechanisms only in periphery without prior injury-related stress (Sung et al., 2004). However, it is interesting that soma hyperexcitability can involve both cAMP and cGMP signaling in the context of peripheral tissue injury (Lewin and Walters, 1999). Under different types of injury-related stress such as CCD, inflammation or dissociation, the two pathways are linked to excitability mechanisms and persistently cross-activated in both the soma and at last maintain soma hyperexcitability in a long-lasting manner (Song et al., 2003b, Aley and Levine, 1999, Aley and Levine, 2002, Bolyard et al., 2000, Liao et al., 1999). All these results indicate that the positive effect of cAMP-PKA pathway on sensory neuron excitability requires prior injury-related stress. Given that CCD has been proved to be applicable to this conclusion (Song et al., 2006, Song et al., 2003b), it is of great significance to test whether dissociation, which is a common process during CCD in many studies, has potential sensitizing effect on neuronal excitability.

## 3 Acute dissociation of DRG produces long-lasting hyperexcitability through enhanced responsiveness to cAMP-PKA and cGMP-PKG pathways

It is crucial to test the hypothesis that acute dissociation of DRG (ADD) may cause cAMP- and cGMP-dependent sensory neuronal sensitization and hyperexcitability, as it might imply that this process does not depend on inflammatory cells recruitment, which is relatively slow. Furthermore, this process may require unique molecular mechanisms intrinsic to injured neurons (Ma and LaMotte, 2005, Ambron et al., 1996, Bedi et al., 1998). Previous investigations tested the electrophysiological properties of DRG neuron in dissociated and intact ganglia from sham and CCD-treated rats. The electrophysiological results show that dissociated DRG neurons from sham rats and intact ganglia from CCD-treated rats can produce qualitatively and quantitatively similar short-term changes in neural hyperexcitability, manifested by the electrophysiological properties of RMP, AP current threshold, AP duration, repetitive firing and the incidence of spontaneously active neurons. Such evidence shows that ADD can indeed cause acute cAMP- and cGMP-dependent hyperexcitability similar to that caused by CCD (Zheng et al., 2007). This is consistent with earlier reports concerning other forms of injury-related stress, especially those induced by axotomy in Aplysia model (Abdulla and Smith, 2001, Song et al., 2003a, Stebbing et al., 1999, Gasull et al., 2005, Ungless et al., 2002, Walters et al., 1991, Gallego et al., 1987, Gurtu and Smith, 1988). In addition, agonists of cAMP-PKA or cGMP-PKG pathway could significantly depolarize RMP, lower AP current threshold, increase repetitive firing previously evoked by a standardized test pulse in acutely dissociated sensory neurons, and also enhance their spontaneous activity in contrast with that of dissociated and sham-intact ones without agonist treatment (Huang et al., 2012). All these results suggest that ADD or CCD treatment can increase the electrophysiological responsiveness to cAMP-PKA and cGMP-PKG pathways in DRG sensory neurons, and also indicate that ADD- or CCD-induced DRG somata hyperexcitability and spontaneous activity require continuing activation of cAMP-PKA and cGMP-PKG pathways.

The finding that dissociation itself can enhance neuronal responsiveness to cAMP-PKA or cGMP-PKG pathway can be of great significance, which raise the intriguing possibility that isolated soma may not display the same functional processes occurring in vivo. First, the dissociation process can enhance neuronal electrophysiological responsiveness to cAMP-PKA pathway and may play a role in cAMP-PKA activation-mediated hyperexcitability of DRG somata as reported previously (Rathee et al., 2002, England et al., 1996, Gold et al., 1996, Ingram and Williams, 1996, Lopshire and Nicol, 1998, Evans et al., 1999, Aley et al., 1998, Smith et al., 2000, Cardenas et al., 2001). Second, these results raise the possibility that nerve injury or associated inflammation activates the cAMP-PKA pathway to enhance neuronal responsiveness to stresses associated with dissociation (Song et al., 2003a, Aley and Levine, 1999, Taiwo et al., 1989, Ma et al., 2006), as described in the next section. It has been revealed that stress such as ADD or CCD can give rise to similar hyperexcitable state, which is mediated through continuing activation of cAMP-PKA or cGMP-PKG pathway. However, the mechanisms underlying the similarity between the hyperexcitable state caused by ADD and CCD require further exploration.

## 4 PAR2 activation is responsible for CCD-, ADD- and trypsin-induced cAMP-dependent neuronal hyperexcitability

Studies of the mechanism underpinning the similarity between CCD- and ADD-induced cAMP-dependent neuronal hyperexcitability have been mounting. The key role of protease-activated receptor subtype 2 (PAR2), which belongs to a family of GPCRs upstream of cAMP-PKA pathway, in the similarity has been preliminarily elucidated (Amadesi et al., 2006, Luo et al., 2007). Endogenous serine proteases cleave the N-terminal structural domain of PAR2, unmasking a ‘‘tethered ligand’’ sequence and then activating PAR2 (Luo et al., 2007). The two most well-known endogenous PAR2 activating proteases are trypsin and tryptase. PAR2 is widely expressed in the nervous system, especially in neurons including most primary sensory neurons (Zhu et al., 2005). Studies have shown that PAR2 participates in several pain-related biological processes including neuroinflammation, nociceptive transduction, and associated hyperalgesia (Bunnett, 2006). PAR2 activation is sufficient to prime hyperalgesia and maintain the chronic pain state through a BDNF/trkB/aPKC signaling axis or NF-κB signaling (Bao et al., 2014, Tillu et al., 2015). In addition, β-arrestin binding and activation of ERK was found in DRG following PAR2 activation, suggesting a translational machinery. The versatile functions of PAR2 depend on the release of two pain mediators: calcitonin gene-related peptide (CGRP) and substance P (SP) (Mantyh and Yaksh, 2001, Traynelis and Trejo, 2007, Vergnolle et al., 2001). Together, these findings indicate a crucial role of PAR2 in chronic pain.

Activation of PAR2-induced hyperexcitability of DRG neurons and behavioral hyperalgesia after ADD or CCD in a cAMP-dependent manner has been suggested. Two different forms of injury-related stress-CCD and ADD as well as trypsin treatment-can induce a similarly increased PAR2 protein level in intact large-, medium-, and small-sized DRG neurons. Further, increased PAR2 in all the three categories of DRG neurons after ADD or CCD co-localized with the increased PKA-c subunit, suggesting participation of the activated PAR2 in regulation of cAMP-PKA pathway (Huang et al., 2012). These findings are consistent with those from earlier reports that injury- or inflammation-induced pathological responses require the participation of PAR2 (Dery and Bunnett, 1999). Both PAR2 activation and neural excitement can be marked by elevation of intracellular [Ca^2+^]_i_ (Steinhoff et al., 2000). Transient perfusion of trypsin were found to cause an increase in [Ca^2+^]_i_ in the small- and medium-sized intact naïve DRGs neurons. Further, PAR2 knockdown by siRNA significantly inhibits trypsin-induced elevation of [Ca^2+^]_i_. These findings support the idea that the elevation of [Ca^2+^]_i_ induced by trypsin is mediated partly by PAR2 activation and confirm that trypsin can excite neurons by cleaving PAR2 (Steinhoff et al., 2000). However, [Ca^2+^]_i_ in viable intact DRG neurons that have already received trypsin application was not altered by additional trypsin treatment. A possible explanation is that the trypsin results in a long-lasting and irreversible PAR2 activation with a ceiling effect (Alier et al., 2008). Further, the same trypsin treatment fails to change [Ca^2+^]_i_ in CCD- or ADD-DRG neurons and the unresponsiveness to trypsin in these neurons shares the similar pattern with that in the intact DRG neurons pretreated with trypsin or PAR2 knockdown. All these evidence supports the notion that injury-induced stress, including CCD, ADD and trypsin treatment, results in DRG neuron hyperexcitability similarly through activation of PAR2. As trypsin is commonly introduced during DRG dissociation to digest tissue extracellular matrix and conduct cell isolation (Huang et al., 2012, Mantyh and Yaksh, 2001, Traynelis and Trejo, 2007), it is rational to postulate that trypsin-mediated PAR2 activation contributes at least partially to the expression of hyperexcitability in acute dissociated DRG neurons. Multiple studies have confirmed that trypsin gives rise to primary sensory neuronal hyperexcitability through cleaving and activating PAR2 (Steinhoff et al., 2000, Sambraus, 1975), the same way PAR2 activating peptide leads to the hyperexcitability of DRG neurons (Alier et al., 2008, Kayssi et al., 2007).

The CCD-, ADD-, or trypsin treatment can increase cAMP concentration and PKA activity in DRG and the activation can be reversed by co-treatment of PAR2 antagonistic peptide (PIP) or knockdown of PAR2 with siRNA, which indicates that both CCD and ADD treatment promote PAR2 activity and result in activation of the downstream cAMP-PKA pathway. Further electrophysiological studies have shown that trypsin and PAR2 agonistic peptide (PAP) can induce hyperexcitability in intact DRG neurons from naïve rats, such neural hyperexcitability can be inhibited by co-treatment with a trypsin inhibitor SBI, or PAR2 antagonistic peptide PIP. However, hyperexcitability induced by trypsin or PAP could not be suppressed by post-treatment of SBI or PIP. Interestingly, post-treatment of AC inhibitor SQ22536 can significantly suppress PAP or trypsin-induced neuronal hyperexcitability. These results support the idea that PAR2 may mediate cAMP-dependent DRG neuron hyperexcitability.

These studies strengthen the notion that the acute dissociation itself may cause cAMP-dependent DRG neuron hyperexcitability. Injury-induced PAR2 activation may mediate the activation of cAMP-PKA pathway, which may then mediate neuronal hyperexcitability following CCD treatment producing inflammation (Ma et al., 2006) and direct nerve injury (Song et al., 2003b, Song et al., 2003c). PAR2 and trypsin could be serving as promising targets for the relief of neural hyperexcitability and behavioral hyperalgesia after nerve injury and similar stresses.

## 5 Activation of cAMP-PKA and cGMP-cGKI pathways in DRG and the spinal cord contribute to the development of bone cancer pain

Severe and unbearable pain caused by bone cancer, primary bone sarcomas or more often distant osteal metastases of bone secondary tumors, happens in over 50% of patients with bone cancer and does not have good response to the currently available analgesics, thus life quality of the patients are severely damaged (Breivik et al., 2009). Most of those terminal patients had already missed the optimal chance to receive radical surgery or multidisciplinary therapies (MDT) (van den Beuken-van Everdingen et al., 2007, Breivik et al., 2009). Therefore, there is an urgent need to better understand the underlying mechanisms of bone cancer pain and look for more effective analgesic strategies. Considerable evidence suggests that mechanisms of cancer pain include nerve injury-induced neuropathy, inflammation, and other unique and yet unknown components (Goblirsch et al., 2005, Ghilardi et al., 2005). The crucial roles of cAMP-PKA and cGMP-cGKI (cGMP–dependent protein kinase I) pathways in DRG and the spinal cord have been primarily identified in bone cancer pain. A significant increase in PKA-RII and PKA-C mRNA, but not PKA-RI mRNA in DRG, coupled with elevation of cAMP concentration and PKA activity in DRG and spinal cord are seen lasting progressively in a long period of time after tumor cells implantation (TCI treatment inducing bone cancer and cancer pain). These long-lasting patterns of molecular changes are corresponding to the painful behaviors observed in TCI rats. TCI treatment results in an increased concentration of cGMP in DRG and activity of cGKs in DRG and the spinal cord, as well as an increased level of cGKI messenger ribonucleic acid and protein in DRG (Zhu et al., 2016, Liu et al., 2014). These findings suggest that cAMP activation in DRG and the spinal cord and activation of cGMP-cGKI pathway in DRG may contribute to the development of bone cancer pain. Spinal administration of inhibitor of cGMP-cGKI pathway can suppress TCI-induced behaviorally expressed mechanical allodynia and thermal hyperalgesia as well as hyperexicitability of DRG neurons (Liu et al., 2014). Repetitive spinal administration of the PKA inhibitor in early- and late- phases following TCI significantly delays or attenuates TCI-induced thermal hyperalgesia and mechanical allodynia. This provides direct evidence that activation of the cAMP-PKA pathway in DRG and spinal cord may mediate the development of bone cancer pain (Zhu et al., 2016). cAMP-dependent DRG neuron hyperexcitability (Huang et al., 2012) may be mediated by the phosphorylation of sodium and calcium channels (Rathee et al., 2002, England et al., 1996, Blackstone et al., 1994, Fraser and Scott, 1999). In conclusion, these studies suggest important roles of cAMP-PKA pathway in the development of bone cancer pain and may provide new strategy of cancer pain treatment by targeting cAMP-PKA pathway.

Bone cancer in human as well as in experimental animals induced by TCI brings about significant bone destruction, which is specifically characterized as loss of medullary bone, remarkable erosion of the cortical bone with additional full-thickness unicortical bone loss. After treatment with the inhibitors of cAMP-PKA and cGMP-PKG pathways, the bone destruction can be greatly prevented or alleviated (Wu et al., 2007, Son et al., 2010). In addition, radiotherapy can remarkably relieve TCI-induced bone destruction and bone loss, which is indicated by the decreased bone destruction score and increased bone structure integrity (Zhu et al., 2014, Zhu et al., 2016).

Radiotherapy is a treatment using ionizing radiation to control or kill malignant cancer cells. Accumulated clinical evidence shows that radiotherapy can produce long-lasting analgesic effect in about 95% patients with advanced tumors (Lin and Ray, 2006, Rades et al., 2010, Chow et al., 2007). Thus, radiotherapy is considered not only an approach to treating bone cancer, but also a strategy for pain relief. However, mechanisms underlying the analgesic effects of radiotherapy in bone cancer remains unclear. Given the evidence that the activation of cAMP-PKA pathway in DRG and the spinal cord plays important roles in bone cancer pain, studies have shown that radiotherapy can relieve bone cancer pain through inhibiting the activation of cAMP-PKA pathway in DRG and the spinal cord (Zhu et al., 2016). A single dose of X-radiation (6 Gy) can produce a significant, weeks-long inhibition of TCI-induced thermal hyperalgesia and mechanical allodynia in TCI rats. In addition, radiotherapy can remarkably relieve TCI-induced bone destruction and bone loss, reflected in decreased bone destruction score and increased bone structure integrity. This phenomenon is in accordance with the earlier report that high-dose radiotherapy can lead to apoptosis in some of the cancer cells or inhibit their activation, growth development and infiltrating bone destruction, which further alleviates and delays the destruction of bone structures and bone loss (Goblirsch et al., 2005, Roodman, 2004). Radiotherapy can also notably reduce TCI-induced increased level of PKA-RII mRNA and PKA-C mRNA in DRG and downregulate cAMP level and PKA activity in DRG and the spinal cord. All these findings indicate that radiotherapy may alleviate bone cancer pain, at least partly, through suppressing the abnormal activation of cAMP-PKA pathway in DRG and the spinal cord.

## 6 Additional mechanisms underlying contributions of cyclic nucleotide signaling to chronic pain

Besides CCD, ADD and TCI treatment, roles of cAMP-PKA pathway has been studied in several other pain models, including partial sciatic nerve ligation(PSNL) (Liou et al., 2007), diabetic neuropathic pain(DNP) (Feng et al., 2017), spinal cord injury (SCI) (Bavencoffe et al., 2016), inflammatory pain (Hucho et al., 2005) and endometriosis pain (Ding et al., 2017). Activation of cAMP-PKA is reported to participate in several upstream or downstream pathways that are related to neuropathic pain. As for upstream signaling, for example, PGE2 in DRG promotes the expression and release of nociceptive mediator BDNF and IL-6 in cAMP-PKA-dependent way (Cruz Duarte et al., 2012). Lysophosphatidic acid (LPA) signaling is pro-nociceptive after PSNL treatment through continuing up-regulating components of cAMP–PKA pathway (Lin et al., 2012). Spinal ephrinB-EphB signaling-related nociceptive information also involves the activity of spinal PKAca and CREB (Zhou et al., 2015). G-protein coupled receptor 3 (GPR3) (Ruiz-Medina et al., 2011), cannabinoid receptor (CB) (Xu et al., 2010), parathyroid hormone 2 (PTH2) receptor are also involved in the development of neuropathic pain through cAMP-PKA signaling pathway (Xing et al., 2003, Sullivan et al., 2007, Inceoglu et al., 2008, Gu et al., 2015, Zhou et al., 2017). As for downstream signaling, cAMP-sensitive component of inward current I(h) and cAMP-related action potential firing crucially contributes to neuropathic pain mediated by hyperpolarization-activated cyclic nucleotide (HCN)-modulated ion channels (Emery et al., 2011). The binding of cAMP can induce conformational modulations in HCN channels, thus regulating the voltage-dependent gating of the channels. HCN channels gain increasing attention recently for their potential as therapeutic targets for neuropathic pain (Tibbs et al., 2016) and four family members HCN1, HCN2, HCN3 and HCN4 have been identified in mammalians. With abundant expression in primary afferent neurons and a determinant role in pain intensity, HCN1 and HCN2 are relatively well-studied isoforms in pain (Tu et al., 2004). In CCI rats, HCN1 and HCN2 are distributed proximal and distal to the injury site on the sciatic nerve. Blockade of HCN channels by specific inhibitors alleviate chronic pain caused by the peripheral injury and inflammation, possibly through the inhibition of I(h) (Jiang et al., 2008, Richards and Dilley, 2015, Smith et al., 2015). In SNI model, persistent firing of layer II/III prefrontal pyramidal neurons was observed after the peripheral nerve injury, which was suggested to be facilitated by the cAMP modulation of HCN channel activity (Cordeiro Matos et al., 2015). The hyperexcitability of pyramidal neurons is considered to contribute to the pathogenesis of chronic pain (Blom et al., 2014). EPAC1 is recognized as a cAMP sensor and can potentiate Piezo2-mediated mechanotransduction in DRG and contribute to nerve injury-induced mechanical allodynia (Eijkelkamp et al., 2013). EPAC1-mediated sensitization of mechanosensor Piezo2 may also contribute to the persistent inflammatory pain, which can be inhibited by G protein kinase 2 (GRK2) through direct phosphorylation of EPAC1 and suppression of the EPAC1-Rap1 signaling (Singhmar et al., 2016). Inhibition of EPAC signaling by low level GRK2 in nociceptors following peripheral inflammation is reported to mediate a transition from acute to chronic pain (Wang et al., 2007, Wang et al., 2018, Eijkelkamp et al., 2010). In addition, PKC, a critical second messenger in sensitization towards mechanical stimulation in both neuropathic pain and inflammatory pain, can be stimulated by EPAC in IB4+ neurons (Hucho et al., 2005).

cGMP-PKG signaling, however, remains controversial because of its complicated roles in the development of chronic pain. Many studies have suggested roles of cGMP-PKG signaling in primary nociceptors responses to various peripheral nerve injuries and chronic pain. Activation of Type I alpha PKG (PKG-1α), an isoform of PKG downstream of cGMP signaling and highly expressed in primary sensory neuron, has been considered to induce long-term hyperexcitability (LTH) in DRG and enhanced spinal synaptic plasticity, both of which are important in the development of chronic pain (Lorenz et al., 2014, Gangadharan et al., 2017). Retrograde transport of PKG to DRG was observed after nerve crush and inflammation, suggesting that PKG could be a limited positive axonal signal in nociceptive neurons (Sung et al., 2006). Inhibition of PKG-1α was found to be an effective strategy to attenuate chronic inflammatory and osteoarthritic pain in rats (Sung et al., 2017). Opioid receptor-mediated antinociception may involve the activation of cGMP signaling and such activation may result in the opening of ATP-sensitive K+ channels and the relief of formalin-induced spontaneous pain (Florentino et al., 2015). B-type natriuretic peptide (BNP)-induced antinociceptive effects of inflammatory pain caused by CFA is also considered to be mediated by PKG-dependent opening of Ca2+  activated K+ channels (Zhang et al., 2010). It is suggested that upstream activators of cGMP-PKG and downstream effects brought by PKG might determine the anti-/pro-nociceptive effects. However, more investigations are required to explain such complexity.

## 7 Conclusions

**Fig. 1:**
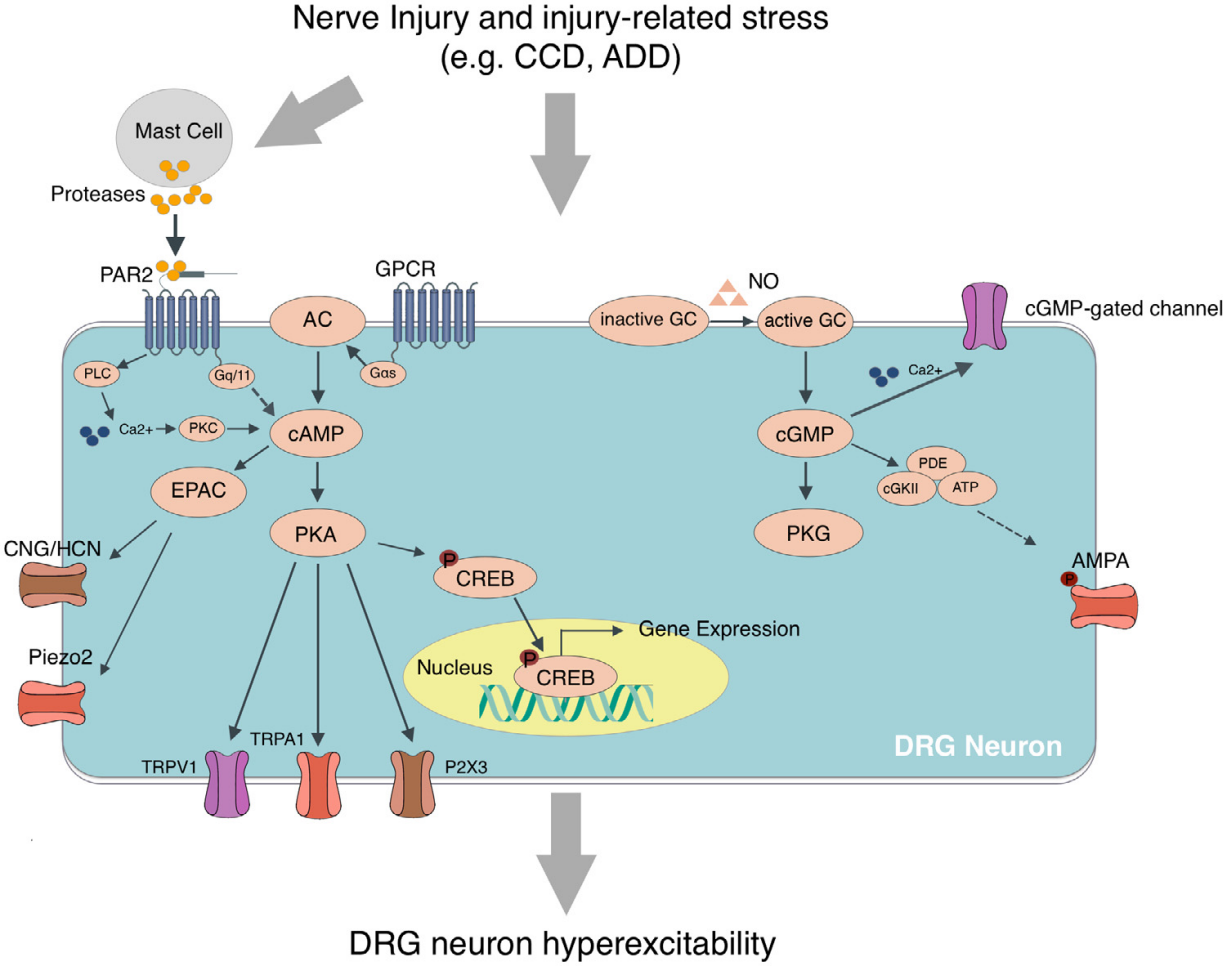
Schematic representation of the roles of the cAMP-PKA and cGMP-PKG pathway in DRG neuron hyperexcitability after peripheral injury or injury- related stress including chronic compression of DRG (CCD) and acute dissociation of DRG (ADD). The activation of PKA-dependent cAMP-PKA pathway may be mediated, at least partly, by PAR2 activation. PKA-independent cAMP-EPAC and cGMP-cGKII pathways also contribute to the neuronal hyperexcitability following peripheral injury.

Activation of cAMP-PKA and cGMP-PKG signaling pathways is crucially important in the development of sensory neuron hyperexcitability after nerve injury and trypsin-assisted dissociation of DRG (See Fig. 1). Such activation of cAMP and cGMP signaling may be mediated by the PAR2 receptor activation following nerve injury and trypsin treatment. cAMP-PKA signaling in DRG and the spinal cord is involved in the development of bone cancer pain. Cyclic nucleotide signaling also contributes to pain due to diabetic neuropathy, spinal cord injury, inflammation, etc. through activation of HCN channels and EPAC. These studies enrich our understanding of the roles of cyclic nucleotide signaling in sensory neuron excitability and the chronic painful conditions and provide new therapeutic insights through both mechanistic and pharmacological elucidation.

## Funding statement

This work was supported by National Nature Science Foundation of China [Grant Numbers 81320108012 and 81671086].

## Conflicts of interest

The authors declare that there is no conflict of interest regarding the publication of this paper.
